# Plants for Extreme and Changing Environments: Domestication, Evolution, Crop Breeding and Genetics

**DOI:** 10.3390/plants13212975

**Published:** 2024-10-24

**Authors:** Freddy Mora-Poblete, Eliemar Campostrini

**Affiliations:** 1 Institute of Biological Sciences, University of Talca, 1 Poniente 1141, Talca 3465548, Chile; 2 Ecophysiology of Tropical and Subtropical Crops, Northern Rio de Janeiro State University, Campos dos Goytacazes 28013-602, RJ, Brazil; campostenator@gmail.com

## 1. Salt Stress Resilience: Germination and Physiological Responses in Crops and Weeds

In this Special Issue, researchers investigated the genetic, physiological, and biological mechanisms that enable plants to thrive in challenging environmental conditions. The original research articles, along with one review article, provide valuable insights into several critical subtopics, including drought and salinity stress tolerance, heavy metal resilience, crop breeding for environmental adaptation, and the role of microbial interactions in rehabilitated soils. Collectively, these studies highlight the potential of advanced technologies such as pangenomics, machine learning, and plant–microbe symbiosis to enhance crop resilience and sustainability in the face of climate change and environmental degradation.

Salinity is a major abiotic stress that limits plant growth and crop productivity, particularly in regions facing water scarcity and soil degradation [1]. Understanding how plants respond to salinity is critical for developing sustainable agricultural practices. In this context, the studies by Nikolić et al. [2] and Ali et al. [3] investigate the challenges of salinity stress on plants, focusing on a widespread weed, *Datura stramonium* L., and *Zea mays* L., a key cereal crop.

The study by Nikolić et al. examines the germination response of *Datura stramonium* under varying levels of salinity (4, 8, 12, and 16 dS/m) and pH (1–9) across two temperature ranges (15–25 °C and 18–30 °C). The findings reveal that *D. stramonium* demonstrates remarkable germination potential even under extreme salinity, with 51.2% germination at higher temperatures (18–30 °C) and significant tolerance to acidic conditions, only showing substantial reductions at pH 2. This adaptability highlights the potential challenges for agricultural management, as *D. stramonium* could thrive in saline soils and compete with crops.

In contrast, Ali et al. focus on enhancing salt tolerance in maize through the application of *Enterobacter cloacae* PM23, a plant growth-promoting rhizobacterium (PGPR). The study shows that inoculation with this bacterium significantly improves maize’s resilience to salinity, evidenced by enhanced antioxidant defense mechanisms, the increased accumulation of osmoprotectants such as proline, and improved physiological traits, including photosynthetic efficiency. Specifically, *E. cloacae* PM23 exhibited multi-stress resistance, producing beneficial compounds like indole-3-acetic acid and exopolysaccharides, which collectively enhance plant growth and mitigate oxidative stress under salinity conditions.

Together, these studies provide valuable insights into how both weeds and crops cope with salinity. Nikolić et al. highlight the adaptive germination capabilities of a resilient weed, while Ali et al. demonstrate the potential of biotechnological interventions, such as PGPRs, to bolster crop tolerance. These findings are crucial for developing effective strategies in managing salinity in agricultural systems, ensuring sustainable crop production amidst increasing environmental challenges.

## 2. Drought Stress Adaptations: Enhancing Crop Productivity Through Genetic and Agronomic Approaches

Drought is a critical environmental factor that significantly impacts crop yield and global food security, making the development of drought-tolerant crops a priority for sustainable agriculture [4,5]. In this context, the studies by Ballesta et al. [6] and Leite et al. [7] explore modern techniques that emphasize the importance of combining genetic insights with traditional breeding to develop crop varieties better adapted to water scarcity. Ballesta et al. employ a machine-learning approach using foliar spectral data to classify genetically distinct subpopulations of spring wheat grown under contrasting water regimes (rainfed and fully irrigated). Their research demonstrates that this innovative classification system achieves high accuracy, with a Convolutional Neural Network (CNN) correctly assigning 92% of cultivars in irrigated conditions and 93% under rainfed conditions. This method facilitates the effective management of wheat genetic resources, enabling breeders to preserve and utilize subpopulations that are adapted to drought conditions.

In a complementary study, Leite et al. assess the agronomic traits of 50 popcorn inbred lines under both water-limited and irrigated conditions across two crop seasons. Their analysis highlights significant reductions in key traits such as grain yield, popping expansion, and nitrogen balance index under drought stress. By leveraging multivariate analysis, the study identifies several promising popcorn lines that maintain high productivity even in water-limited environments, with lines L294 and L688 excelling in popping expansion and L691 and L480 showing superior grain yield.

Both studies underscore the importance of combining genetic insights with agronomic traits to develop crops better adapted to water scarcity. Ballesta et al. illustrate the potential of machine learning in managing genetic diversity, while Leite et al. provide actionable insights into the selection of drought-tolerant genotypes. Together, these findings contribute to addressing the critical challenges posed by climate change and advancing sustainable agricultural practices.

## 3. Coping with Heavy Metal Stress and Rehabilitated Environments: Genetic Mechanisms and Microbial Interactions

Heavy metal contamination and degraded environments present significant challenges to plant growth and food production, necessitating the development of crops and species that can thrive under these adverse conditions [8]. In this context, the studies by Raja et al. [9] and Nascimento et al. [10] investigate how plants adapt to such environments through genetic mechanisms and interactions with soil microbes.

The study by Raja et al. analyzes the genetic behavior of *Solanum lycopersicum* (tomato) germplasm under wastewater irrigation. The research reveals that certain genotypes, particularly RIOGRANDI, exhibit enhanced tolerance to heavy metals by limiting their translocation to the aerial parts of the plant. Genetic analyses indicate that both additive and dominant gene actions contribute to heavy metal tolerance, with a significant increase in cumulative additive variance for flowering and fruiting traits in wastewater-irrigated plants. This work highlights the potential for breeding tomato varieties that can flourish under wastewater irrigation, a practice increasingly vital in regions facing water scarcity.

In contrast, Nascimento et al. focus on the adaptive mechanisms of *Dioclea apurensis*, a native plant from the Amazon, in rehabilitated minelands. The study examines proteomic changes and microbial interactions that allow this species to manage oxidative stress and nutrient deficiencies in degraded soils. The findings indicate that *D. apurensis* displays specific proteomic responses that enhance its survival in challenging conditions, as well as an ability to associate with various beneficial soil microorganisms, which supports its establishment in these environments.

Both studies emphasize the importance of understanding genetic and microbial mechanisms in plants growing under stress, providing insights that could inform strategies for crop production and soil management in contaminated or degraded landscapes.

## 4. Pangenomics for Crop Adaptation to a Challenging Environment

Finally, one review article was included in this Special Issue. Petereit et al. [11] explored the impact of crop domestication and breeding on shaping wild plant species into modern high-yield crops suited for key agro-ecological regions. However, with climate change threatening crop productivity, the review highlights the urgent need for agriculture to adapt to ensure future food security. Crop wild relatives, which thrive in more diverse environments than domesticated crops, harbor a genetic diversity that may offer vital traits for resilience in changing climates. The review emphasizes the potential of pangenome analysis to uncover the genomic basis of stress tolerance in wild species, providing crucial insights for breeding climate-resilient crops. By identifying and utilizing these untapped genetic resources, breeders can transfer resilience traits to domesticated crops or even re-domesticate wild species, combining environmental adaptability with high productivity to meet the agricultural demands of the future.

## 5. Conclusions

Altogether, this Special Issue provides significant insights into how plants adapt and respond to extreme and variable environments, contributing to our understanding of crop resilience and adaptation strategies. Several studies in this collection, including those focused on drought and salinity stress, underscore the importance of genetic and physiological mechanisms for enhancing stress tolerance. Notably, the role of spectral data in classifying wheat cultivars and the identification of drought-tolerant popcorn genotypes represent promising advances for breeding programs aimed at developing climate-resilient crops.

While these studies advance knowledge in crop adaptation, challenges remain, particularly in fully integrating genomic data and biotechnological tools into practical breeding applications. As high-throughput technologies such as pangenome analysis, machine learning, and microbial profiling become more widely adopted, future research will likely provide deeper insights into plant adaptation strategies. These efforts will be critical in addressing the agricultural challenges posed by global climate change and environmental degradation.
